# A community-embedded approach to increasing the health literacy of Aboriginal children in a regional area: processes of co-design and local implementation

**DOI:** 10.3389/fpubh.2024.1355603

**Published:** 2024-03-06

**Authors:** Phillip Good, Rebekah Grace, Catherine Kaplun, Janet Conti

**Affiliations:** ^1^Transforming Early Education and Child Health (TeEACH), Translational Health Research Institute (THRI), Western Sydney University, Penrith, NSW, Australia; ^2^School of Psychology, Western Sydney University, Penrith, NSW, Australia

**Keywords:** Aboriginal health, child-centred, co-design, community-embedded, implementation

## Abstract

**Purpose:**

This research explores the implementation of a child-centred, co-designed, community-embedded program called ‘Young Doctors for Life’ (YDFL). YDFL is designed to improve health and wellbeing outcomes for Aboriginal children in the middle childhood years. Focus is given in this paper to the processes of program adaptation of the YDFL to ensure local cultural relevance, drawing on the experiences and perspectives of children, parents, schoolteachers, and the implementation team.

**Method:**

Two focus groups with program stakeholders were convened. The first group consisted of three members from the local Aboriginal implementation team, and the second group comprised two members of the program design team. Children (*n* = 22) and schoolteachers (*n* = 2) participated in semi-structured interviews. Parent survey data (*n* = 16) were also collected and included. The data was analysed, guided by the five elements of implementation as outlined in the Hexagon Implementation framework (Capacity; Fit; Need; Usability; Support; and Evidence), which served as *a priori* themes.

**Results:**

YDFL provides a promising example of how programs can be adapted with and for Aboriginal communities to support child health. Successful adaptation and implementation of this program required a co-design approach engaging program designers and the local implementation team. Community collaboration was also essential to identifying and addressing local community goals and aligning new programs with local service and cultural contexts.

**Conclusion:**

Health programs to support positive child outcomes are more likely to be successful when they share their focus between the risks and challenges within a community, and the positive, protective factors that can be leveraged to support children to flourish. Stakeholder engagement and community leadership are necessary to achieve meaningful program adaptation and implementation in Aboriginal communities.

## 1 Background and introduction

The ‘Young Doctors for Life’ (YDFL) program is run by the Malpa Project for Australian Aboriginal^1^ children in their middle childhood years. It is a child-centred health intervention program designed to support improved child health outcomes in Aboriginal communities.

A particular focus in the program is health literacy and the role that children can play in advocating for positive health behaviours across their communities. The YDFL program is implemented only by invitation of community leaders and requires the formation of a local Aboriginal leadership and implementation team. Before the program commences with children, the local team assesses and adapts the program to embed local cultural elements and blend Western health practices with traditional knowledges. Adaptation of a program in response to local culture and needs is argued within the research literature to be critical to program sustainability and successful outcomes (1). In the Australian context, there is a strong call for Aboriginal people and communities to be at the centre of the service decision-making processes (2). This paper describes the local process of YDFL adaptation and implementation in one regional community, and how the program was received and experienced by local stakeholders.

Australian Aboriginal communities experience significant health inequities, similar to those of other Indigenous peoples in colonised nations (3, 4). These inequities are evident from childhood. For example, In Australia, Aboriginal peoples experience the highest documented burden of Rheumatic Heart Disease in the world (5), 90% of Aboriginal children living in remote areas have some form of Otitis Media (6) and there is a trachoma endemic within regional, rural, and remote Aboriginal communities, with Australia bewilderingly remaining the only high-income country reporting such widespread trachoma (7). The observation that, overall, health inequities for Aboriginal people continue despite significant government investment (2) leaves little doubt that there is a need to re-think the approaches and paradigms that underpin health service provision for Aboriginal children and their communities.

Reflective of a difficult socio-political and historical landscape, a discussion of the factors that underpin current health inequities experienced by Aboriginal children and their communities are beyond the scope of this paper. However, it is important to acknowledge that Australian Aboriginal communities experience high levels of intergenerational trauma as the result of strong state interventions, including: state-sanctioned child removal policies between 1909–1969 (8); the ongoing over-representation of Aboriginal children in the child protection system (9); high rates of incarceration (10, 11); and the ongoing struggles for the recognition of Aboriginal peoples as the custodians of their lands (12). An understanding of the complexity of this history and the impact of ongoing systemic racism is essential to redressing health inequities and honouring the resilience and self-determination of Aboriginal communities (13).

In addition to historical factors, research documents the complex array of social determinants associated with poor health outcomes in Aboriginal communities, including inadequate and overcrowded living conditions, and low levels of educational attainment, employment, and income (13). In contrast, the social determinants associated with positive health outcomes for Aboriginal communities include cultural identity, connection to kinship, knowledge and beliefs, language and participation in cultural activities, access to traditional lands and caring for country (14).

There is a growing number of researchers who argue that the investment in health programs has not achieved the anticipated improvements in health outcomes because of the lack of community co-design and consultation, which has rendered programs meaningless to communities and unsustainable (15, 16).

A major criticism of Aboriginal health programs is that they are frequently imposed on communities by service organisations who lack an understanding of the communities they intend to assist, who decide what problems exist and develop solutions without engaging in respectful communication or shared decision-making with the community (2, 17).

### 1.1 Culturally meaningful program implementation

This paper aims to explore the implementation of a child-centred health intervention program which aims to improve the health literacy of Indigenous children, and to encourage them to be health advocates within their community. An implementation science approach underpins this study, which employs a case study approach to examine the local adaptations that are necessary to ensure the program is locally meaningful, and the perceived impacts of the program. The voices of key stakeholders, including children, parents, teachers and the local implementation team are privileged in this study. The study seeks to contribute to the body of research on the importance of child-centred, co-designed, community embedded approaches to implementation to support meaningful outcomes and sustainability. When Aboriginal people are asked about their aspirations for health service improvement, focus is often given to the centrality of relationships rather than a focus on clinical disease. For example, Smith and colleagues interviewed 60 Aboriginal people of the Maningrida community in Arnhem Land (15). The participants spoke of the Maningrida construct of *urrutu*, which is the relationship between an individual, their kin, and their Country. They described wanting a public health care service built on trust and relationships, delivered by language-congruent Aboriginal health professionals guided by panels based on language groups. Bulloch and colleagues conducted case studies of three Aboriginal health and wellbeing services (18). All three services emphasised the importance of a community-driven approach that employed holistic health and person-centred practices for the delivery of effective services. For all three organisations, strengths-based approaches were inseparable from a community-driven, holistic service design (18).

Apart from being good and respectful practice, the leadership and engagement of Aboriginal people and communities in decision-making regarding the provision of health initiatives is reinforced by the United Nations Declaration on the Rights of Indigenous Peoples (19). Guided by the purposes and principles of its Charter, the United Nations upholds the right of Aboriginal families and communities to retain shared responsibility for the upbringing, training, education, and wellbeing of their children. Article 23 of the Charter includes the right of parents and the community to be actively involved in developing and determining health and social programs and to administer such programs through their own institutions. Social processes such as community co-design, deliberative decision-making, and participation can support public health improvements by engaging people in an outcome-oriented learning and capacity-building exchange (20). This process of evidence-informed decision-making is paramount when working in culturally diverse communities, as cultural assumptions that sit behind intervention programs must be challenged (21).

The international implementation science literature provides helpful frameworks for reflection on the appropriateness of intervention programs for diverse community contexts and the processes of place-based adaptation (22, 23). One useful example, the Hexagon analysis tool requires that decisions about program implementation in any community are guided by consideration of six essential indicators (24).

- Capacity to implement—are there staff who are appropriately qualified to implement the program or strategy? Is this program or strategy sustainable within this community?
- Fit with current initiatives—does the program align with community priorities? How does it fit within the existing service network and community values?
- Need—are the issues being addressed by the program seen as significant by all stakeholders and supported in data indicating need?
- Usability—is the program or initiative ready to be implemented? Does it need further adaptation to be meaningful and culturally appropriate in the local context?
- Support—is training and professional development related to this program or practice readily available? Is training culturally sensitive? Does it address issues of race equity, cultural responsiveness, or implicit bias? What is the source of training and professional development?
- Evidence—what is the strength of the existing evidence of effectiveness for this program or strategy?

The RE-AIM framework (Reach, Efficacy, Adoption, Implementation and Maintenance) is another widely used implementation science tool (25). RE-AIM guides program evaluations to consider factors beyond efficacy, and to incorporate measures and evaluation designs that broaden the range of factors beyond clinical measures only (26, 27). In recent years, RE-AIM has been used in evaluations of health promotion programs in Australian Aboriginal contexts (26). For example, it was employed in the evaluation of the Deadly River Mob (DLM) program, a peer-driven, incentivised health promotion program to reduce hepatitis C among Aboriginal and Torres Strait Islander peoples (26). The program was found to produce positive effects in a culturally safe way, with the employment of frontline staff seen as key to fostering community trust and engagement (28).

For the purposes of this study, the Hexagon analysis framework was employed because it gives stronger focus to decision making around implementation and adaptation which is aligned with the purpose of this research, where-as RE-AIM is more strongly focused on evaluation and measures of success (24, 29).

### 1.2 The Malpa project and the young doctors for life program

The Malpa Project is a not-for-profit organisation that is the home of the core YDFL program. YDFL offers the same information and core materials to all communities who invite them to deliver the program. However, a co-design process is undertaken with local Aboriginal Elders, to adapt the program, and shape the program content to be appropriate and responsive to community customs, knowledges, and needs. The passing of knowledge from Aboriginal Elders to young people is acknowledged as an important tradition in Aboriginal communities (30, 31). Respecting this sharing of knowledges, YDFL incorporates the sharing of the old and new ways with children to develop their role as health ambassadors and active agents of change within their own lives and communities. The program designers believe *‘there is real wisdom in the old ways’* and see the program as an avenue through which to *respect* traditional cultures and healing practices while also sharing Western health knowledge. The language of the local people is incorporated into the program for each site wherever possible and is evident in the program names. For example, the program is called Tjitji Doctors in Alice Springs, and Bubup Doctors in Melbourne (32).

The Young Doctors for Life program commenced in 2012 with 12 students at Alice Springs in the Northern Territory of Australia. The program has grown so that 4,100 children across Australia are expected to have completed the program by the end of 2024.

Malpa has conducted their own internal evaluations. These evaluations, combined with a body of strong anecdotal evidence, point to many positive outcomes for the program, particularly as this relates to child and parent school engagement, improvements in health behaviours, and child sense of connectedness to culture.^2^ There is a need to establish a formal evidence base for the effectiveness of the program.

### 1.3 The current project

The adaptation and implementation of the YDFL program in one regional community is reported in this paper. The perspectives and experiences of the local Aboriginal implementation team, the original YDFL design team, the participating children, their parents, and school staff are presented and discussed. The evaluation was guided by the following research questions:

1. What was the implementation process for the YDFL program.
2. Who was involved and what were the actions necessary to ensure that the program was relevant and culturally meaningful within the local community context?
3. What were the challenges associated with the adaptation for the cultural context, while maintaining core program components and the fidelity of the established YDFL program?
4. What were the experiences and perspectives of children, parents, and school staff, regarding the implementation of the program and its relevance to the local context?

## 2 Methods

A qualitative case-study design using focus groups, interviews and surveys was employed to understand the processes and experiences of program adaptation and implementation in one regional site. Aboriginal children were recruited if they had completed YDFL training within 2 years of the study commencement date, with no child excluded. All parents and schoolteachers of the children taking part in the study were also asked to be involved in the research. No exclusion criteria were applied to adult participants.

A qualitative case study design was employed because we were interested in understanding the nuanced and context driven nature of program adaptation and implementation. The research literature argues that programs are most effective when they embed local knowledge, take into account environmental and place-based issues, and are culturally meaningful (18). While this argument is strong conceptually, there are very few descriptions within the literature of how this happens and when it has happened successfully. We hope to contribute to the literature by capturing this process in one community, privileging the experiences of the stakeholders directly involved. Future research will explore this process in other communities. We will look to identify the key support mechanisms of meaningful adaptation and the impact on child, family and community outcomes.

Questions used to guide the focus groups were based on the key implementation indicators of the Hexagon analysis tool (24), as described earlier. The larger program of research also examined program efficacy; however these findings will be reported in a subsequent paper. The focus of this paper is program adaption and implementation.

### 2.1 Aboriginal consultation

The research team was invited by the Malpa Project to undertake independent research and evaluation of their Young Doctors for Life (YDFL) program. The Malpa leadership team connected the researchers with the group of Aboriginal Elders who were leading the program delivery in the case study site. The researchers adhered to the Australian Aboriginal Health & Medical Research Council’s five key criteria for conducting research with Aboriginal children and communities, including: demonstrating the overall positive benefit for Aboriginal health; ensuring Aboriginal community control of the research; establishing and sustaining cultural sensitivity; reimbursing participation costs; and enhancing Aboriginal skills and knowledge.

The first author, a doctoral student who conducted the field research, worked collaboratively and over a sustained time frame with the regional Aboriginal leaders of YDFL prior to commencing the research to support the development of trust and shared understanding of the method and appropriate community engagement strategies. The building of trust was supported by the fact that the field researcher was also a person with an Aboriginal family heritage. Following consultation with the local Aboriginal Medical Service, an Aboriginal reference group was formed and a formal invitation to commence the research was received from the regional YDFL project coordinator.

### 2.2 Ethics statement

This study was approved by the Aboriginal Health & Medical Research Council Human Research Ethics Committee (Reference number: 1561/19). This approval was recognised by the Western Sydney University Human Research Ethics Committee (WSU HREC). NSW State Education approval was obtained prior to interviewing children at their school. Privacy note: pseudonyms are used for children’s names.

Ethical child researchers are reflexive in creating conditions where children have agency and are supported to share power as much as possible (33). Specifically, the researcher acknowledged and sought to address the implications of the child’s perception of the adult researcher as unduly powerful as this may inhibit their free responses or make the child uncomfortable. The field researcher was always introduced to a child by an Aboriginal Elder. He was presented as a person with Aboriginal family heritage who was interested in what children thought about health issues, and specifically the YDFL program. He explained that he was not a teacher within the school system. The field researcher used child-friendly language and humour, dressed casually, and was relaxed to avoid replicating a teacher’s role and to decrease power imbalances. He explained he was a researcher who saw children as experts on their lives and cared about what they think. The field researcher acknowledged the respect of children and adults following his invitation from the Aboriginal community to undertake the research.

### 2.3 Participants

This study engaged five groups of participants.

*Group 1: Members of the local implementation team*: the local implementation team comprised three participants who were all Aboriginal and responsible for the adaptation and implementation of YDFL in their region. The Aboriginal Elder in this group had primary school qualifications and all three participants had experience with face-to-face teaching and formal qualifications in community support.

*Group 2: Malpa program design team:* two senior members of the program design team participated in the project. Both participants had business and education qualifications.

Group 3: *Children:* the study involved two cohorts of children. Cohort 1, or the Retrospective Cohort, consisted of 14 children, aged 9–12 years (8 females and 6 males), who had completed the YDFL program within the last 2 years. Cohort 2 consisted of eight children, aged 7–11 years (5 females and 3 males), who were interviewed before and after program participation. No non-Aboriginal children were involved.

Group 4: *Parents*: 16 parents of children from Cohort 1 or 2 participated in the research.

Group 5: *School staff*: two schoolteachers of children from Cohort 2 participated in this research. One schoolteacher was Aboriginal, and the other was non-Aboriginal.

### 2.4 Recruitment and data collection

A face-to-face focus group was conducted with the local Aboriginal implementation team. Participants were provided with the focus group questions in advance of the session with assurances they could raise any issues that were most important to them without feeling restricted by the questions. Examples of questions included:

To what extent have you adapted the program specifically for the needs of the community?

Would you see the core program being easily adapted for other regional communities?

The program design team also participated in a one-off face-to-face focus group guided by questions informed by the Hexagon analysis tool.

Due to COVID lockdowns and the associated travel restrictions, as well as a series of natural disasters in the region including flooding, the original research plan to conduct a mixed methods pre and post design study with children participating in the program for a year was not feasible. Instead we interviewed children who had previously completed the program (Cohort 1), recruiting them with the assistance of local project champions. Nineteen interview questions were used to scaffold their reflections on program participation and any sustained learnings and impact. Later, when community access improved, we recruited another cohort of children (Cohort 2) to the study and proceeded with the original pre-post design, including gathering qualitative experiential data and measuring child outcomes. Only the findings that relate to program implementation are reported in this paper.

Parents of the participating children from both cohorts were invited to complete a hardcopy survey in their own time and return it to the field researcher. The survey was comprised of open-ended questions asking for their reflections on the program and its impact on their child and family.

School staff participated in individual, face-to-face semi-structured interviews to capture their views on the YDFL program and any observed program impacts on participating children. Dates of data collection for each participant group are shown in Table 1.

**Table 1:** Participant data-gathering dates.

Participants	N	Age range (years)	Data collection: Pre—YDFL	Data collection: Post YDFL
Child cohort 1	14	9–12		June – December 2020
Child cohort 2	8	7–11	April 2021	November–December 2021 (disrupted by Covid lockdowns) May 2022
Parents	16		April 2021	November–December 2021 (disrupted by Covid Lockdowns) May 2022
Schoolteachers	2			May 2022

### 2.5 Analysis

All participants were given the opportunity to review the transcripts from their interviews and to add or subtract information. A thematic analysis was undertaken. The field researcher and one other research team member developed the initial coding framework using the six elements of the Hexagon analysis tool as *a priori* themes and identifying the unique data segments that captured how each participant constructed meaning based on personal experiences of the YDLF program implementation and adaption. Memo writing recorded researcher insights that afforded more depth and complexity to the coding process. Codes were refined and relationships with and between codes developed to identify themes and subthemes. The researchers have reported back to the local community and stakeholders, with plans to provide a final report after the completion of the study.

## 3 Results

**Figure 1:**
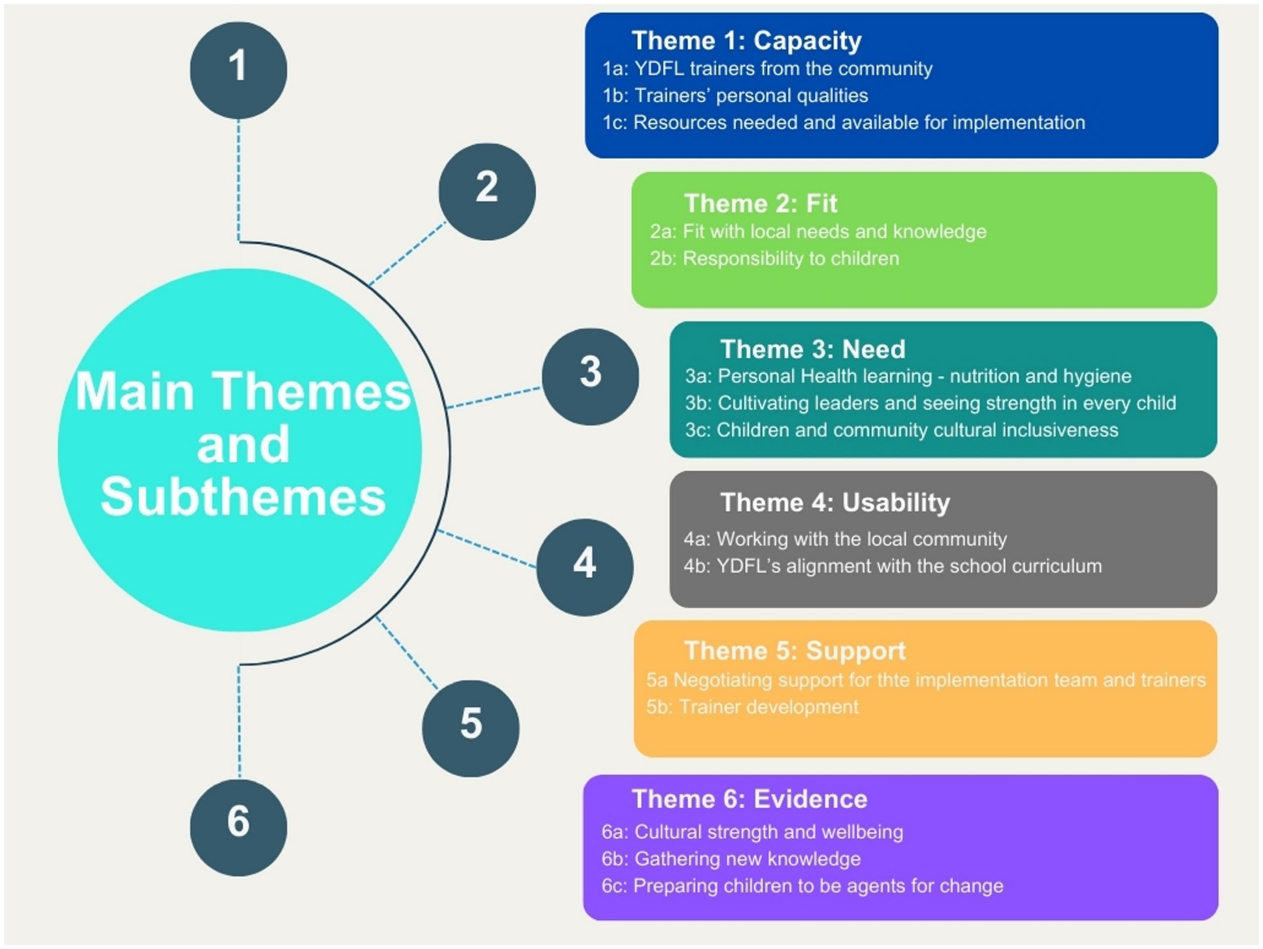
Themes and subthemes.

The perspectives, experiences and health education approaches employed by the local implementation team in establishing the Malpa YDFL program in a regional community were explored. Data were analysed and organised according to *a priori* themes: Capacity; Fit; Need; Usability; Support; and Evidence. Each theme and their subthemes are discussed. Themes and subthemes are shown in Figure 1.

### 3.1 Theme 1 capacity

The research participants saw capacity as relating to both personal and available community resources. Their views were captured across three subthemes relating to the importance of local trainers with specific qualities, and adequate resourcing.

#### 3.1.1 Subtheme 1A: YDFL trainers from the community

To have the YDFL trainers drawn from the local community was seen as imperative by all participants in the focus groups. A program designer said: “Another thing about the capacity to implement going beyond skill, one is that we require that one of the people is Indigenous, at least one preferably two.”

When the implementation team was asked how important it was to be part of the community when the program was being implemented, they affirmed its importance, expressing their views based on their own experiences of living in the community.

Yes, it gains trust, if people know – with the Indigenous community, if they do not know you and you were someone who flew in to do the same thing – they do not know you – they do not know what your motives are – they just assume you are another government worker or whatever. But if you are a member of the community and they have seen you out there. They are relaxed. (Implementation team member 1).

The importance of having Aboriginal trainers was also clear in the narratives of the children, who felt safer sharing their views when the adults were also Aboriginal.

It, sort of, made me a bit more confident to … made like everyone in our group to be, kind of, a bit more confident – and to, I guess, speak out because – because most of us or some of us, they feel like they cannot speak out because of, like, our colour and stuff. And even though that there’s no racism at our school – that we know of, we still feel like that we cannot – do stuff, so that sort of helped us. (Irene, aged 11 years).

The children’s schoolteachers also saw the leadership of local Aboriginal people as important to learning for the children, incorporating learning about their own heritage.

The [local name] Doctors is one of the best things [for learning culture and heritage]. I learnt things that I did not, you know. And I always say to kids that—we are learning—we [teachers] are learning as well because we do not know everything. (Aboriginal schoolteacher).

#### 3.1.2 Subtheme 1B: trainers’ personal qualities

The personal approach of the individual trainers was seen as important to successful program implementation.

… we want them [the trainers] to be imaginative and we want them to be able to put their own unique stamp on their project. So they need to have a capacity to try and to think differently in terms of how they engage the kids and how they connect with the school and their community. So they need to be good communicators. They need to have good interpersonal skills. Uh, they need that capacity like a good uh, a good sports coach to be able to energise the room, keep kids on focus and make them feel that somebody is genuinely interested in the kids themselves and what they are doing and have an idea about what they might become. (Program designer 1).

The children valued trainers that injected a lot of fun and supported them to find their voices. The routine yarning circles^3^ and generally yarning with the children throughout the 15 week program was particularly valued. As Lucy (aged 11 years) commented “I like how we sing the song on the yarning circle mat. Yeah. Because we were all doing it, yeah. Yeah, having fun*.”*

A sense of fun, energy, and ability to empower children were seen as the most important qualities for the trainers, beyond their Aboriginality.

#### 3.1.3 Subtheme 1C: resources needed and available for implementation

When asked if the YDFL was sustainable in their regional area, the implementation team Elder told the facilitator that the team receives funding from the Malpa Project for each program site implementation. It was a challenge to make the funding stretch, and the local team needed to make decisions and work to a budget to prioritise the needs of the children attending each YDFL program, as the Elder explained:

I have cut back on the spending so much, but I have increased it on nutrition—I have increased the funds and how much we spend because I want them to taste good food not cheap food—more variety, so you have to spend more. (Implementation team member 1)

The Elder pointed out that the program is sustainable because of the small budget. However, in her view, if there were no funds available, the program would continue with the dedicated volunteers who were part of the program delivery: “You could deliver this with volunteers— you do not need a big budget—enough to buy the snacks and things like that*.”*

### 3.2 Theme 2: fit

In the discussion of the core curriculum, program designers shared that they needed to be aware of the core curriculum, however there is a need to be adaptable to meet the needs at the community level, and in terms of the different health regulations across the states of Australia. Adaptability was critical.

#### 3.2.1 Subtheme 2A: fit with local needs and knowledge

The adaptations do not happen at the level of head office, they happen in a co-design process with local implementation teams in response to the needs of communities: “We do not do the adaptations, no—the community creates their own, yeah— they are the ones adapting the program to make it relevant” (Program designer 1).

The implementation team stressed the importance of adapting the YDFL program to children’s differing needs and knowledge depending on the context. One implementation team member, referring to one of their innovations, commented, ‘We are the only people who do the song and the pledge - and I love it…it makes more ownership for the kids’.

Another implementation team member took pride in the program being locally driven and owned:

The essence of this thing [program] is that that it is a home-grown … all the programs have commonalities but are really very, very different. [We] construct the ways of delivering according to what they [community] need and I think one of the real strengths of the program is this localisation… [by way of] it’s structural flexibility… so community adaptation [is] right from the get-go and gives the local community ownership. (Implementation team member 1).

The children described being taught things that were responsive to their needs, evidence of the fit of the program as this relates to what was important to the children.

I felt like they, uhm, have helped me a lot for the past years, because like I have got bullied at school and all that, but I think they [YDFL trainers and peers] help me with all of that and now, I do not really get bullied. And they helped me with being safe, and healthy, and not smoking and all that. (Mila, aged 10 years).

#### 3.2.2 Subtheme 2B: responsivity to children

The implementation team explained that some children could not read and write or may have had learning disabilities. Nevertheless, they reported how they were responsive to children in an ongoing way across program delivery, so the challenges the children were facing did not stand in the way of their training as health ambassadors and becoming potential leaders for their peers and the wider community:

You do [adapt] a lot and you find out what is happening to children…as adults we are adapting ourselves to the child, not having the child adapt to us, …one time I will do this and next week this—I’m [often] slightly trying to change and adapt the activity to the—sort of, need for the kids. (Implementation team member 3).

One of the children interviewed mentioned that he had a disability and found mainstream schooling difficult. When asked what he remembered most about attending the YDFL training he replied, “Probably how we, uh, sat in a circle on the mat and sang songs and talked, yeah” He went on to describe how he did not feel like he had a disability when he was with the group like this. In their responses, the parents said that their children’s socialisation skills had improved following involvement in the YDFL program and indicated this was because of the trainer’s responsiveness to their children. One parent spoke about how isolated her child had been, and how the YDFL implementation team had supported the friendship connections that were formed within the group:

… it’s helped with like my – with like people who have gone to [the Young] Doctors because we can talk about – like all this stuff. And they also talk – they also talk about like friendships and stuff and that helps us, uhm, I guess with feelings and stuff for friendship and – Yeah. (Irene aged 12).

Parents described witnessing their children’s increased confidence to engage with others, including making new Aboriginal and non-Aboriginal friends. The program designers perceived that the trainers developed the ability to skilfully adapt the program in response to immediate need as they gained more and more hands-on experience. As one program designer said:

It is much as possible, being experiential and hands-on … they learn by implementing the program. They are discovering the boundaries that they may have thought constricted them, that only exist in their mind, and if they go off on the slightly strange tangent from our point of view, we want them to know that we are not looking over their shoulder. We are happy to support them and go wherever that might go, even if it sits slightly outside where we were thinking. (Program designer 2).

The program designers expressed belief in the values, skills, and expertise of each local program implementation team to deliver an adapted and tailored program to their community because they knew the children best. Programs were therefore centred on the children’s needs.

### 3.3 Theme 3: need: empowering children as ‘young doctors’

The heath challenges for Aboriginal children in the area were well known to all of the participants, and so there was a strong sense of shared need. The delivery of the program centred on key areas of need. The interview data described the growing ability of children to address those needs and become leaders within their own communities.

#### 3.3.1 Subtheme 3A. personal health learning—nutrition and hygiene

Two priority issues for the case study community centred on nutrition and hygiene, and so the program was adapted to ensure emphasis on this form of personal health learning. The participants provided several anecdotes in which they could see the impact of their program in this respect. For example, children influenced their parents to purchase nutritious food, such as choosing grain bread instead of traditional white.

The implementation team described how they managed their budget to ensure they purchased wholesome food for their sessions with the children that focused on eating healthy foods. Following is an example of a local adaptation aligned with perceived local needs.

I have increased how much we spend because I want them to taste good food, not cheap food … You are validating the kids, and you are validating that they are human beings. (Implementation team member 1).

We do the food tasting—you try the different cheeses— ‘ohh I’ve never had this before’— ‘well, go get your mum to buy it’—and some would just stand there and keep eating— ‘like can I take it on the bus?’—and they will take the packet home to show mum and dad. (Implementation team member 1).

The trainers also described discussing sensitive matters with the children relating to personal hygiene in an age-appropriate and non-offensive way. The implementation team, knowing the financial disparity in their regional area, and being aware that some families were struggling and not able to afford things like washing machines, worked to normalise the situation. For example, they created a game to help children take responsibility and look after their clothes:

We did this one in relay teams where one washes [an item of clothing], drops it and the next one rinses it, one pegs it up and the next one puts it on an ironing board and the next one [folds it]. The children are very competitive, and they are saying, ‘come on come on’—and the boys—the competitiveness about the whole thing—learning and they love the game. For two weeks in a row, we had to play the game because they just loved it. But they were learning that clothes do not get left in the basket … They were learning that they could go into the bathroom and wash it if they do not have a washing machine. [It’s about] learning how much water to wash your clothes [in] and taking responsibility to be respectful of your clothing and of your parents who have to buy them. (Implementation team member 3).

These extracts exemplify two important implementation components that support positive outcomes from the perspectives of the implementation team, namely: the value of games in facilitating behaviour change without shame or embarrassment; and the importance of cultivating a sense of personal responsibility. Having fun while learning new things and experiencing health education through games was a positive experience for the children. Extracts from children supported this perspective.

[I liked] playing and learning about new stuff and playing things that you never played before. (Jade, aged 9 years)

Then we’d do like some games and stuff—but they were also still educational which was really good. (Denis, aged 11 years)

Making fun things every day…We made our own playdough…Yea, and we put it into—we made a stress ball. [What are the best things?] The activities. They made them more fun than they usually are. (Ivy, aged 11 years)

The schoolteachers agreed that the playful and fun nature of program implementation was impactful in supporting the learning of the children and facilitating their engagement.

… You cannot have education boring. You are going to have education fun. It’s going to be cultural, you know, it’s a good way of learning, you know, in a fun, cultural, respected way, we believe anyway…I believe. (Aboriginal Schoolteacher)

I think it’s got a good balance because they do their talk time, and their workbook time, and then they also have their outside playtime, and then their food time, they love the food. So, no, I think it’s a very good balance of everything, really. (Non-Aboriginal Schoolteacher.)

The implementation team was successful in marrying the need for information and health learning, with the need for connection and fun.

#### 3.3.2 Subtheme 3B: cultivating leaders and seeing strength in every child

In the case study area, the implementation team felt there was a need for local leadership and saw their role as supporting children to become local health leaders.

If you give the kids the tools to lead, to be their own leaders and adults, they will step up to the plate. (Implementation team member 1).

It was particularly edifying for the implementation team when they saw child leadership demonstrated, and this encouraged them to continue prioritising supporting the children in the development of this skill. For example, when the school held a graduation ceremony for the kindergarten children, the YDFL implementation team were amazed to see the recently graduated ‘young doctors’ volunteer to lead the ceremony: ‘They just stood up and said we can do this’. The implementation team strongly perceived that the involvement of the older children in the YDFL program gave them the confidence to support the younger children and be leaders and role models for them.

The implementation team described the selection process for children attending the YDFL program. In some instances, the school staff nominated the children; on other occasions, the selection of participants was made in consultation with the implementation team. The latter was considered to be ideal because this mitigated against the usual practices of school staff to select the children who they felt deserved a reward for their good behaviour.

The teachers do not look at the kids as individuals before they pick them. He will be alright … he is naughty, so he will not be doing it. I do think it is good that the good kids do it because it is a reward for them, but the kids who muck up and stuff probably need it more. They are a bit lost and at some schools when we say [to the children] you are a leader, they say, ‘no we are not, [name] is a leader—we could not be a leader.’ We tell them that you were picked because you have something good to give—we build their self-esteem. (Implementation team member 3)

The implementation team advised that selecting participants who were potential leaders and health ambassadors required insight and understanding. As an example, an implementation team member related the following instance where leadership potential was not immediately obvious:

We had two boys at [school name] who we had at a community play group … We knew the mum and family, trouble kids who were always suspended. So, when we went to their school, we said that we want those boys. They [school] had not thought of them because they were looking at those who were trying to be good kids. But we had those boys, and we had a guest speaker who also worked with the boys elsewhere, and he said that he had never seen them sit quietly [before]. (Implementation team member 2).

The implementation team’s intent was to empower children. The YDFL program is not taught in a didactic manner but is activity-based with a cultural focus. The implementation team felt that the children were often more comfortable in that space than in their mainstream classrooms, which was then reflected in their behaviour when engaged in YDFL activities.

There is scope to meet the individual needs of each child while maintaining fidelity to the core program, which is an important aspect of implementation. The mix of children and the ability to adapt to their needs was considered important by the program designers.

I mean, most of our programs seem to choose a mix of people who are likely to be leaders, and sometimes there are kids who are currently leaders in a negative way, but they [trainers] want to pick up on their leadership ability and turn [them] around. We have some programs where more than 50% of kids are in out-of-home care. (Program designer 1).

The program designers spoke of the absence of a tiered reward system, with all Young Doctor graduates receiving the same certificate:

We do not believe, in any type of class… we do not do any of that …you know competition is a good thing [and] rewarding excellence…we [just] want the kids to feel all proud and all in this together…and they do, and this is often the first certificate they have ever received… their parents feel proud… if you can empower one you can empower more than one. (Program designer 2).

Parents spoke positively of the social cohesion, equity, and confidence building within the YDFL program their children attended. For example, one parent said:

She gives things more of a go now than before, it has lifted her self-confidence. (Parent 3).

#### 3.3.3 Subtheme 3C: children and community cultural inclusiveness

While the YDFL program has been designed for Aboriginal children, non-Aboriginal children are also welcome to attend in the spirit of inclusiveness. The implementation team and the program designers recognised the need to promote activities that may unite both Aboriginal and non-Aboriginal children and families from the regional area. “They [non-Aboriginal children] are coming from exactly the same socioeconomic group as the Aboriginal kids” (Program designer 1).

An Aboriginal child may invite a non-Aboriginal “buddy” to join them in the YDFL program. This was reported as a way of achieving inclusivity in the region.

Then when they [non-Aboriginal] come to the [YDFL], they are so proud that they are part of the group … and we push it and say you are really special, and you have a role to play you have an important position and job [to tell people] … and they boast about it. They tell their non-Indigenous parents. (Implementation Team Member 1)

One implementation team member spoke of her wish to eliminate the ‘black areas and ‘white areas’ of the regional township. The Elder of the implementation team (Team Member 1) spoke of her initiatives to improve a park in the town where people could visit and undertake activities or just rest there.

So, to try and fill that gap, I went on the board of [name] park [with] the bush tucker garden and I am the chair. So, by helping the community I am helping the [YDFL program]. So, once the plants [re-grow], it has been run down for a while, (then) running school programs with the council with other groups for teenagers and parents [can occur]—the town is getting right back into weaving. I have been doing weaving at [name] Park, but I have to pass it on to the kids. And I hope they will come to a weaving group with us. (Implementation Team member 1).

These initiatives correspond to comments made regarding connecting to culture and understanding their environment. As one parent stated, “I think it is a great program for the kids, Aboriginal and non-Aboriginal, I think it helps the kids to connect to culture and understand culture while learning how to look after themselves and their environment.” *S*uch comments were reinforced by another parent who suggested changes that could help such as “more info about the program, [and to have] parents more involved, if they want to, and more Elder involvement.”

### 3.4 Theme 4: useability

The YDFL program’s usability relies on how successfully it is embedded within the target community. Being considered part of the local Aboriginal community was described by the implementation team as crucial to their efforts in the successful delivery of the YDFL program. The implementation team felt that their community membership and acceptance paved the way for program implementation by building trust and a spirit of collaboration. The local trainers’ engagement with their own community was considered a distinct advantage by the Sydney-based program designers. This theme includes two subthemes that highlight usability through the adaptability of the YDFL program.

#### 3.4.1 Subtheme 4A: working with the local community

The program designers rely on the local implementation team to embed the program within the community by working closely with the people from the community.

We do not know all those communities and there’s no way we can ever know all those communities. So, we need those people on the ground to be able to do that for us…and they really do rise to the occasion. (Program designer 1)

Furthermore, the implementation team engages other local health service providers in the program to raise children’s awareness of the local community health services available to them. This initiative included representatives from local services such as the dental service, a hearing service, a smoking prevention program, and health promotion educators who promote hygiene practices to prevent the spread of bacteria and viruses.

A frequent response from parents was that they observed the YDFL as a fun way for their children to learn about hygiene practices. They often commented on ‘Mr Germ’, a character in the program, and his related presentations, which reinforced good hygiene practices in a fun way. As one parent said:

Mr. Germ and the black light, and teeth brushing [made my daughter] independent and clearer of her own environment [so she was able to understand], mask, cross contamination.

The relevance of the YDFL program became even more obvious to parents when the COVID-19 virus spread to regional and more remote areas of the state. The YDFL program was instrumental in supporting the children to understand the importance of personal care procedures.

[Our child taught our family] Better hygiene, hand hygiene—showing family how to wash hands [during COVID]. (Parent 5)

[My son] did not complain about washing hands and requirements of Covid 19. It prepared [him] for good hygiene practices with him regularly washing and sanitising his hands. (Parent 11).

#### 3.4.2 Subtheme 4B: YDFL’s alignment with the school curriculum

The program designers referred to YDFL’s compatibility and enrichment of the NSW school core curriculum.

I think part of the reason that is welcome is that it delivers on the core curriculum, and so, it’s a different way of doing what they are required to do anyway. In a way, [it] is a lovely, sort of, an enrichment into the school but is not a bolt on the side. It’s right in the middle. I think that’s absolutely fundamental. If it’s an add-on, it will not-will not be picked up. (Program designer 1).

The implementation team worked closely with the school to ensure that the program complemented the curriculum and the timing of lessons to align with what the school is working on.

It’s great because it fits straight in with our PDHPE syllabus [Personal Development, Health, and Physical Education], to teach about healthy relationships, healthy lifestyle, everything—and being good leaders and what good friends were about, so it all fits in perfect, yeah, and our-our kids love it. (Non-Aboriginal Schoolteacher).

Both schoolteachers observed the children’s enthusiastic response to new learnings from the YDFL program, and the supportive relationship the YDFL trainers had developed with the children. They felt that participation in YDFL had a positive influence on the children’s engagement with the wider school curriculum.

### 3.5 Theme 5: support

It is the role of the program designers and the regional program coordinator to ensure training support for the YDFL implementation team and trainers. This theme is discussed in two subthemes.

#### 3.5.1 Subtheme 5A: negotiating support for the implementation team and trainers

The program designers, cognisant of the YDFL trainers’ positive influence on the children, have developed support processes for their trainers during the delivery of the program. Nevertheless, focus group participants described early tensions between the program design team and trainers arising from the requirement for trainers to submit regular reports on the YDFL program. Trainers reported “finding the task overly complex and time consuming.” To resolve these tensions, the participants in the focus groups described negotiation processes to balance the trainers’ support needs and the accountability mechanisms required by the Malpa Project head office.

One of the difficulties the design team encounters is the geographical distance between themselves and the regional trainers. Regular contact enables the design team to become aware of the implementation team and trainers’ concerns. As a result of negotiation between both parties, the process has become more streamlined, catering for both the educational reporting requirement, addressing the trainers’ concerns, and ensuring that local Aboriginal voice is privileged.

The implementation team also established a mutual support system from within the broader YDFL community to share ideas and resources: “that’s what we need…support from other groups… this resource works for us here and so on… what works for here may work for you.” The program designers took notice and organised an electronic chat group to facilitate this networking. However, this was not taken up by other trainer groups. The implementation team continues to explore options to create communication networks between sites on suitable social media platforms.

#### 3.5.2 Subtheme 5B: trainer development

Close liaison is required between the program designers and the local implementation coordinator to support ongoing implementation and adaption in line with local need. The program designers talked about how they kept in touch on a regular basis with the implementation team and responded promptly when advise was sought:

They will frequently email me. Sometimes ring me or text me asking for advice about how to go about something…that is much more informal, but it is at least fortnightly contact with every program. The ongoing capacity development tends to happen through that [process]. (Program designer 1).

The program designers reported offering the YDFL trainers formal education courses (e.g., accreditation through TAFE), but to date there had not been strong interest in this offer. There was acknowledgement that the trainers were strongest when their local knowledge was honoured, and when they engaged in the process of experiential learning.

One implementation member described a process of ‘research mindedness’ in which the implementation team was constantly asking questions about what was needed, what was working, and how the program could respond.

We do research, we do a lot of our own research. We look at things and bring it to the table, discuss it and say, ‘do you like this or this.’ We have regular meetings and talk about what we will do next week and what we will bring to the table. (Implementation team member 1).

### 3.6 Theme 6: evidence

In the Hexagon tool, the implementation element ‘Evidence’ is largely centred on previous research and practice evidence to support the decision to implement a particular practice or program in the first place. In our case study, ‘evidence’ was interpreted to mean ongoing observations to see whether children were responding to the program and adapting the program in response. The four subthemes under this heading, therefore, capture the implementation team’s observations of positive change that fuelled their practice.

#### 3.6.1 Subtheme 6A: cultural strength and wellbeing

In the delivery of the YDFL program, culture or cultural strength is not always spoken of directly. Culture is often taught through sharing information and stories, fun activities, and yarning circles. Below are examples of responses from children when they were asked if the YDFL program helped them feel strong in their culture.

I reckon it did, yeah, even though that it wasn’t about like culture, you still felt connected … being there with, like, basically with a few people, and like—being with kids, uhm, that, like, are Indigenous too and just learning about the same stuff. That’s, like, the best part. (Irene, aged 12 years).

Not power. Uhm, responsibility, uh, keeping people safe, uh, if you—if there’s like a boulder that people still like get through, that you can move it. And uhm, if you have like a problem you have to like—uh, you have a problem like you like to move something, you can move it. (Len, aged 9 years).

YDFL provided an opportunity to strengthen friendships among Aboriginal children and facilitated the power that comes with joining together as a community to “keep people safe” (Len, aged 9 years).

Children’s answers referred directly to cultural strength and the importance of their cultural identity for wellbeing.

Uhm, like connecting to your culture like always—like when you are not—when you are weak with your culture, you are not learning about your culture. And when you are strong, you are always learning about it, and you have Elders talking to you. It’s sort of like knowledge when you are strong. (Irene, aged 12 years).

Observations from the implementation team around the importance of strengthening children’s connections with each other led to an expansion of their work into the broader community, such as encouraging children to become part of gardening and cultural groups.

#### 3.6.2 Subtheme 6B: gathering new knowledge

The YDFL program is co-designed for local areas with traditional Aboriginal knowledge holders, Elders invited in by the YDFL trainers, in partnership with doctors, nurses, ambulance paramedics, dentists and nutritionists—whoever holds the knowledge that will empower the ‘young doctors’ to become health ambassadors. The children described being encouraged to open themselves to new knowledge and experiences. This willingness was evident in the narratives of the children.

We had to learn like—when we like did not wanna try anything out—we had to [try] … Then I said to Auntie Jan, I said, ‘Auntie [trainer], what is that?’ and she said, ‘It’s passionfruit.’ And then she said, ‘Taste it,’ and I said, ‘No.’ And then she opened it and then I said, ‘Ewe, I do not wanna look at that.’ And then I ate it and then it tasted good. (Margo, aged 9 years).

… learning new stuff…and learning like the Aboriginal, uhm, plants. And the berries that you can eat and not. And how to make the paint. I think—I think I remember once they put that on to protect their face from the sun. It like protects their face so they do not get burned when they hunt. Yeah. (Tess, aged 12 years).

The children demonstrated enhanced knowledge of nutrition.

They’ve told us like the, I guess, good and bad food and that sort of changed how I see food. And they tell us like what you can and cannot eat and they—they also introduce us to new foods and salts and like jams and stuff like—uhm, like cultural sort of foods that sort of has changed my diet too. And like—yeah. I guess they are teaching us—what we used to eat, like, back in the day and that. (Irene, aged 12 years).

The children also gained new knowledge of hygiene that shaped their behaviours.

So I would [now] always wash my [hands] before and after I eat dinner and after I go to the bathroom or outside, or I’ve touched the animal. Uhm, I’ve cleaned my room every Sunday, and I wiped down walls and clean my sheets and stuff, to make sure my room is all clean. And I always, uhm, get—I—if I’ve had water sitting in my drink bottle all day, I will refill it at night and put it in the fridge. And I would clean my dishes and fork, knives, and things like that after I’ve eaten, yeah. [Why is it good to wash your hands?] Uhm, so if you are touching things and then someone else goes to touch it, that they do not get your germs, if you have been—uhm, not picking your nose or playing with dirt or something, just so you can keep your germs to yourself and that. So, then people do not—because it’s very important to keep yourself healthy and your hygiene. (Steph, aged 11 years).

The children also demonstrating that they were learning to access the health system.

[If you were really sick] I would probably—I, think we’d go to the hospital straight up, they’d [parents] take me. [Who would help you there?] I think the nurses would see me first—and if something is badly wrong, they’d get a doctor. But if they can figure it out better, they’d give me the medicine I might need and tell my parents to what times and what days to take these. And make sure and come back maybe like two weeks tops to see if I’m doing okay. (Tess, aged 12 years).

The trainers advised that they wanted the program to be rich and stimulating first and foremost and prioritised this above cramming a large amount of information into the program. They chose to use an approach marked by gentle guidance and encouragement to help the children develop skills and gather new knowledge in the areas that were most relevant and appropriate for their community.

#### 3.6.3 Subtheme 6C: preparing children to be agents for change

The schoolteachers witnessed a change in the children who participated in the program as this relates to helping their families and others. The felt that the program supported children to become agential.

[The YDFL] is a really good program, I think. I think it’s helped them. But it’s helped them to help people who need help as well. Do you know what I mean? That sort of carries on….

[the children say] I keep coming to school, learning all this good stuff, and then going home and saying, ‘Mum, that’s no good for you or dad, that’s not—you cannot smoke that or drink that.’ You know, the kids are seeing, you know, both sides of the good and the bad but, yeah. (Aboriginal school teacher).

The schoolteacher interviews in particular gave insight into how the YDFL program had effects beyond the classroom and into their families and communities. The implementation team saw this as evidence that their methods were having an impact and should be continued.

## 4 Discussion

This paper presents findings related to the adaptation, implementation, and participant experiences of the Young Doctors for Life (YDFL) program in a regional Australian Aboriginal community. The participants described a collaborative implementation process involving the national leaders of the YDFL program, the local Aboriginal YDFL program implementation team, the local Aboriginal community, children, and a local school where the YDFL program was delivered. It showcases the factors that are impactful in supporting the delivery of culturally appropriate health services in Aboriginal communities and the importance of locally meaningful program adaptation. Fixsen and Blase (34) propose that the successful adaptation and implementation of a program is a dynamic and ongoing process in response to the context and needs of local children, families, and communities. By way of their lively and energetic responses, the members of the implementation team conveyed their enthusiasm for community engagement in delivering the program. There are key learnings presented in this study that may assist the implementation of other programs intended for children in Aboriginal communities.

The processes employed by the YDFL program implementation team align with what is discussed in the research literature as best practice in culturally meaningful program adaptation, including the importance of: establishing local leadership, building relationships with the local community and program co-design; supporting the participation of children in child health education programs; and bringing together local and cultural knowledges with Western health knowledges (1, 18). Each of these elements is discussed.

### 4.1 Establishing local leadership, building relationships, and program co-design

Aboriginal Elders are vital leaders who enable community ownership and control of health initiatives that benefit their communities. Aboriginal cultural leadership is an expression of cultural values in action. The concept of cultural wellbeing was conveyed by Aboriginal Elders in the study by Cox and colleagues (35) as three interconnected themes: mentoring, cultural healing and balance between the community’s cultural foundation and the delivery of health and service provider programs (35).

With widespread concern that health programs in Aboriginal communities are ‘imposed’ without respectful communication and shared decision-making (2) this study provides an example of one way in which a standing organisation might work effectively with Aboriginal communities. In recent years we have seen a growing body of research that deliberately seeks to shift the power imbalance and ownership of programs, with examples including Australia’s first National Aboriginal and Torres Strait Islander Cancer Framework (1); a drug and alcohol, and mental health comorbidity project in Adelaide, South Australia (36); and the development of a community rehabilitation and lifestyle service for a remote Aboriginal community in Northern Queensland (37). Use of co-design in each of these examples was found to be crucial to merging Western evidence and systems knowledge with local need and inclusion. This is fundamental in fostering ownership and knowledge translation for Aboriginal communities (1). In the current study, community acceptance of the program was evident and perceived to be strengthened by the implementation team who were all active members of the local Aboriginal community. Community collaboration was understood as essential to enable the implementation team to align the program with local community health initiatives and to partner with local stakeholders. This alignment with, and understanding of local supports, was found to build trust between program organisers, facilitators and the community in the development and implementation of the YDFL program. Ongoing health service engagement is supported by respectful community relations, community involvement, and co-design. Sustainability is reinforced when the program implementation team and trainers live in the community, enabling program uptake and ongoing assessment and improvements.

### 4.2 Supporting child participation

This study highlighted the importance of adapting programs to meet individual child needs. A study by Priest et al. (38) reported that Aboriginal children faced greater challenges than children in the general population in the achievement and maintenance of health and wellbeing due to several factors in their social, historical, and political environment. Therefore, essential to children’s needs are programs that understand the local context and seek to ensure equity of access to high quality programs for all children. Child engagement and participation in positive experiences is crucial to counter-acting challenges in their lives and supporting positive outcomes (39).

A strong argument exists in the literature that improved child health outcomes will occur when health service providers listen to the voices of Indigenous children (40) and support them to be agents of positive change. The Secretariat of National Aboriginal and Islander Child Care called on researchers to hear the voices of Indigenous children as they explore domains of wellbeing, namely: safety, health, culture and connection, mental health and emotional wellbeing, home, and environment, learning and skills, empowerment, and economic wellbeing. One way this can be achieved is listening through yarning, the Australian Indigenous cultural form of conversation (41). There is a body of child participatory research that details the creative and arts-based qualitative methods that can be employed to support the engagement of children in research and the sharing of their perspectives (42). The findings from the current paper support the importance of participatory methods and seeing children as active stakeholders with a role to play in shaping program decision making.

### 4.3 Support for trainers

Trainer education was identified within the findings as key to successful program implementation. There were two educational aspects for trainers: firstly support from the program designers and, secondly, support within the implementation team. By providing training to local team members, new insights into the community context were gained and local knowledge was built into the program supporting its effective adaptation to the local context. The implementation team considered the implementation of program elements and offered suggestions to meet the local context before the program was delivered and throughout program implementation.

The importance of trainers’ experiential learning as they engage in the education process has been recognised in previous educational research (43). Peer support and peer-mentoring programs have also gained popularity in higher education and other learning environments. (44). To enhance trainer’s skills the design and structure of a mentoring program should be connected to the program goals (45). A mentoring program design is the key to peer mentoring program success. In the wider implementation of a health intervention, trainers’ support and mentoring will require capacity for implementation costs and the required resources (24).

### 4.4 A framework for Aboriginal child health interventions

Translating research findings into practical outcomes and insights is complex and open to critique, notwithstanding the unpredictable results from theory to practice and the various levels of continuing assessment required (46). The following diagram depicts the complexity of developing health interventions for Aboriginal children and what needs to be considered when designing, disseminating evidence, and implementing the intervention. A successful intervention is not linear; however, it comprises a holistic sum of parts that require continuous evaluation in practice and research.

**Figure 2:**
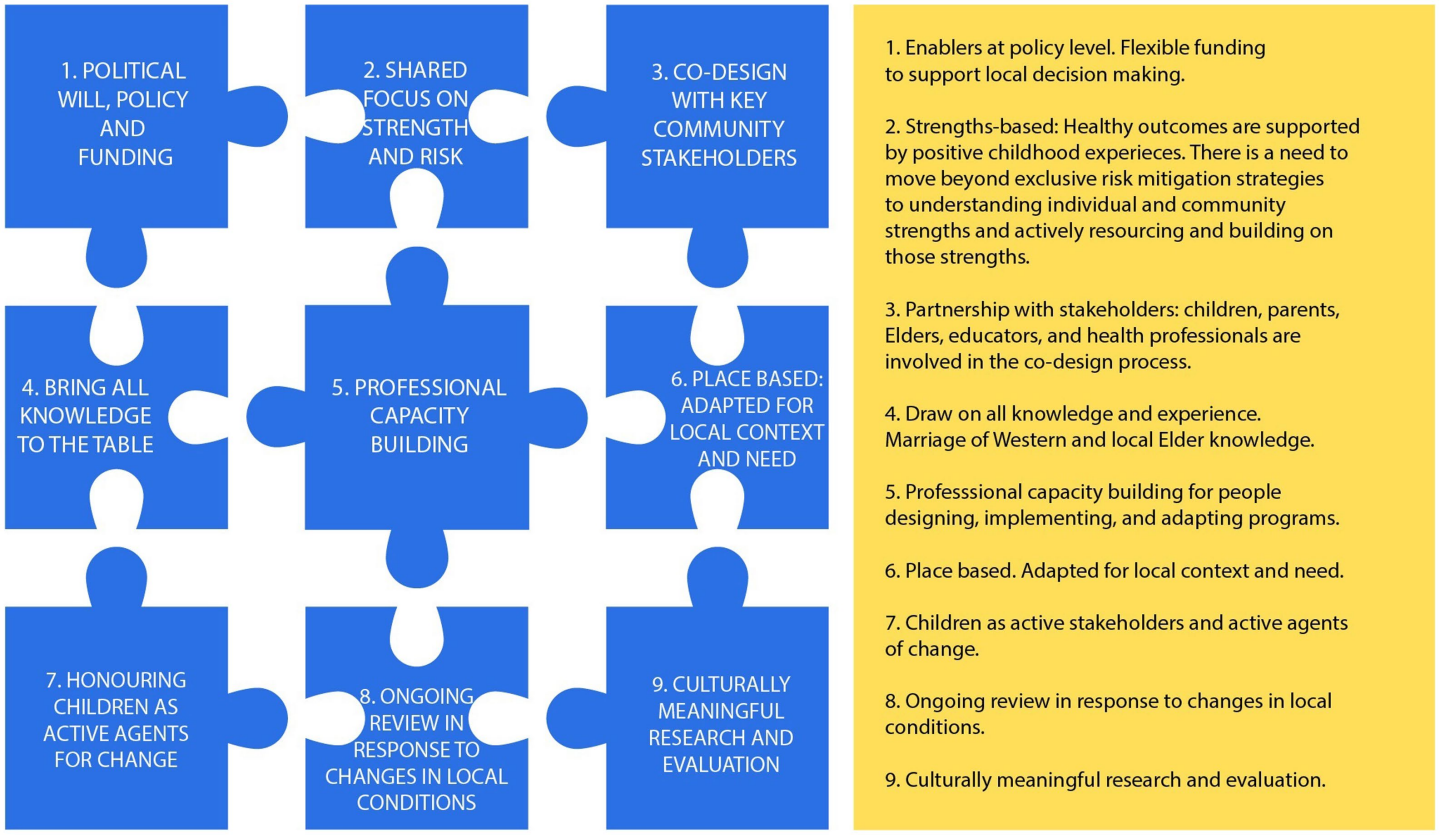
Developing health interventions.

The information in Figure 2 uses the jigsaw analogy to indicate the non-linear conceptual framework of implementation science. Furthermore, each part of the process requires focus for all the parts to come together as an impactful whole-child health intervention. There is a need to review programs that have been implemented to refine and adjust where required over time. The analogy also applies to future research as researchers explore new ways to improve implementation, dissemination and adapting health interventions where Aboriginal children are at the centre of the process.

### 4.5 Future research

Jigsaw pieces 8 and 9 in Figure 2 refer to the full implementation phase of innovation and testing the application of the framework represented by the other pieces. In this phase, team members and educators support and expand the innovation using data for continuous improvement and improved outcomes. Future research will test the application of this framework, both in the expansion of research focused on the YDFL program and in relation to other Aboriginal child health programs. Future projects will include exploring the implementation of the YDFL program in other diverse community settings, examining the long-term effects of YDFL on child and community outcomes, exploring how existing Aboriginal child health programs may be enhanced by engaging with children as active stakeholders and exploring the role of children as active agents of community change more broadly.

### 4.6 Limitations

This research was impacted by state-wide COVID-19 restrictions. For example, it was only possible to conduct the research in one regional community. Access to other regional or remote areas would have provided a more comprehensive study. COVID-19 travel restrictions also prevented the employment of a pre-post design for all of the participating children, and this compromised the rigour of the design to some extent.

There is potential for bias in the program evaluation as the children enjoyed the program activities. Their enjoyment could potentially mean that they overstated the impact of the program. Similarly, there may be biases from employees who believe in the importance of the program and may downplay challenges or drawbacks because they do not want to risk having the program discontinued. The research field officer has Aboriginal family heritage and belongs to an Aboriginal community healing and leadership group. While recognising his positionality provides a unique insight, to reduce any potential bias the analysis and interpretation of the data involved discussions and was conducted in collaboration with the research team.

### 4.7 Implications

The findings of this study have direct relevance to practice for those working to address the systematic differences in health status and outcomes for Aboriginal children. This paper emphasises the importance of participatory, community-driven models, and calls for consideration of the role of children as active agents for positive change. A child-centred approach to program delivery, in which programs are continuously adapted in line with the needs of local children, is vital. Health programs must incorporate aspects of local culture and healing practices as integral to the implementation of intervention programs if programs are to be meaningful, culturally appropriate, and respectful in local contexts. Leadership from local community members, with a foundation of local knowledge and culture, is a critical strength that should be prioritised. This study has shown that programs are strengthened when they build on existing health and community programs, and partner with a broad range of stakeholders across the health, education, social welfare, and community sectors.

## 5 Conclusion

The YDFL program was found to be collaborative in its implementation and delivery of culturally appropriate and child-centred health services in an Australian Aboriginal community and provides an example of locally meaningful program adaptation. Community co-design and collaboration was essential to identifying and addressing local community goals and aligning the program with local health initiatives. The community was an integral part of the program which addressed the needs of their children and complemented existing and accepted health initiatives. Importantly, adaptation and implementation of the program required the recognition of each child’s individual needs and learning requirements. The inclusion of children in this process was fundamental to ensuring the program was fun, educational, appropriate, and relevant to the children’s needs and lives. Community acceptance of the program was strengthened by having the implementation team and trainers as active members of the relevant community as it allowed trust to be built between both entities, which encouraged greater acceptance and buy-in of the program. Successful adaptation and implementation in part required a collaborative approach between the trainers and the children; an approach that focused on growing the healthy, positive, protective factors that already existed in their communities. For this program, as with all health interventions, it was important to consider all aspects of implementation science, and the successful adaptation and implementation of the program, to ensure sustainability and embed ongoing evaluation for continued improvement of the program over time.

## Data availability statement

The original contributions presented in the study are included in the article/supplementary materials, further inquiries can be directed to the corresponding author.

## Author contributions

PG: Conceptualization, Data curation, Formal analysis, Investigation, Methodology, Project administration, Writing – original draft, Writing – review & editing. RG: Conceptualization, Supervision, Writing – review & editing. CK: Conceptualization, Formal analysis, Supervision, Writing – review & editing. JC: Formal analysis, Supervision, Writing – review & editing.

## Footnotes

^1^The words Aboriginal and Indigenous are both used respectfully in this article. The term ‘Aboriginal Australians’ refers specifically to the First Nations people of mainland Australia. When the term ‘Indigenous’ is used in the Australian context, it respectfully refers to both Aboriginal people and the First Nations people of the Torres Strait. There are approximately 150 distinct language and cultural groups of Indigenous people in contemporary Australia, living both on and away from their traditional lands.

^2^ https://www.malpa.org.au/

^3^Yarning circles: A term used by some Aboriginal communities to refer to processes of bringing a group of community members together for the purpose of gathering specific information, in accordance with local community and cultural protocols.
